# Effects of Caffeinated Coffee on Cross-Country Cycling Performance in Recreational Cyclists

**DOI:** 10.3390/nu16050668

**Published:** 2024-02-27

**Authors:** Daniel Trujillo-Colmena, Javier Fernández-Sánchez, Adrián Rodríguez-Castaño, Arturo Casado, Juan Del Coso

**Affiliations:** 1 Sport Sciences Research Centre, Rey Juan Carlos University, 28943 Madrid, Spain; daniel.trujillo@urjc.es (D.T.-C.); javier.fernandezsa@urjc.es (J.F.-S.); adrian.rodriguez@urjc.es (A.R.-C.); arturo.casado@urjc.es (A.C.); 2 Program of Epidemiology and Public Health (Interuniversity), Ph.D. International School, Rey Juan Carlos University, 28943 Madrid, Spain

**Keywords:** stimulant, supplementation, mountain biking, sports performance, dietary supplement

## Abstract

The ergogenic effects of acute caffeine intake on endurance cycling performance lasting ~1 h have been well documented in controlled laboratory studies. However, the potential benefits of caffeine supplementation in cycling disciplines such as cross-country/mountain biking have been rarely studied. In cross-country cycling, performance is dependent on endurance capacity, which may be enhanced by caffeine, but also on the technical ability of the cyclist to overcome the obstacles of the course. So, it is possible that the potential benefits of caffeine are not translated to cross-country cycling. The main objective of this study was to investigate the effects of acute caffeine intake, in the form of coffee, on endurance performance during a cross-country cycling time trial. Eleven recreational cross-country cyclists (mean ± SD: age: 22 ± 3 years; nine males and two females) participated in a single-blinded, randomised, counterbalanced and crossover experiment. After familiarisation with the cross-country course, participants completed two identical experimental trials after the ingestion of: (a) 3.00 mg/kg of caffeine in the form of soluble coffee or (b) 0.04 mg/kg of caffeine in the form of decaffeinated soluble coffee as a placebo. Drinks were ingested 60 min before performing a 13.90 km cross-country time trial over a course with eight sectors of varying technical difficulty. The time to complete the trial and the mean and the maximum speed were measured through Global Positioning System (GPS) technology. Heart rate was obtained through a heart rate monitor. At the end of the time trial, participants indicated their perceived level of fatigue using the traditional Borg scale. In comparison to the placebo, caffeine intake in the form of coffee significantly reduced the time to complete the trial by 4.93 ± 4.39% (43.20 ± 7.35 vs. 41.17 ± 6.18 min; *p* = 0.011; effect size [ES] = 0.300). Caffeine intake reduced the time to complete four out of eight sectors with different categories of technical difficulty (*p* ≤ 0.010; ES = 0.386 to 0.701). Mean heart rate was higher with caffeine (169 ± 6 vs. 162 ± 13 bpm; *p* = 0.046; ES = 0.788) but the rating of perceived exertion at the end of the trial was similar with caffeinated coffee than with the placebo (16 ± 1 vs. 16 ± 2 a.u.; *p* = 0.676; ES = 0.061). In conclusion, the intake of 3 mg/kg of caffeine delivered via soluble coffee reduced the time to complete a cross-country cycling trial in recreational cyclists. These results suggest that caffeine ingested as coffee may be an ergogenic substance for cross-country cycling.

## 1. Introduction

Caffeine (1,3,7-trimethylxanthine) is the most widely consumed psychoactive substance worldwide. Approximately 80% of the world’s population consumes caffeine on a daily basis, mainly because of its psychostimulant properties [1]. Caffeine acts primarily on the central nervous system by binding to adenosine receptors and blocking the action of adenosine, resulting in increased neurotransmitter release and decreased pain perception and fatigue [2,3]. Although caffeine increases physical performance in general, there is, however, a certain inter-individual variability in the response to caffeine intake, depending on factors such as caffeine habituation and time and form of ingestion, among other variables [4]. Caffeine can be naturally found in some plants such as coffee, tea or cocoa, but it can also be artificially incorporated into foods and beverages. Caffeine is most commonly consumed through beverages such as coffee, tea or energy drinks but there are other forms of caffeine administration such as capsules, pre-workout supplements or chewing gum that may be more suitable in some situations, such as exercise [4].

Caffeine has been consistently shown to improve performance in endurance sports [5,6] when ingested at moderate doses ranging from 3 to 6 mg/kg body weight [4]. Particularly, there is plenty of evidence demonstrating the effect of caffeine on enhancing endurance cycling performance [6,7,8]. In cycling trials, caffeine intake within this dose range improves performance in both time to exhaustion trials [9,10] and time trials [7,11]. However, the literature on the benefits of caffeine for endurance cycling performance has two main limitations when it is transferred to endurance cyclists.

On one hand, most of the literature on the effect of caffeine on cycling performance has been tested with caffeine anhydrous via capsules. This is a common experimental procedure as the dose of caffeine administered can be accurately administered in powder form within a capsule for research purposes while avoiding the interference of other substances. However, athletes have a variety of options available when it comes to selecting how to obtain their caffeine dose before exercise [12]. Caffeinated coffee represents a popular manner of caffeine consumption before exercise for endurance athletes [13], particularly for cyclists [14]. Although the amount of caffeine in a cup of coffee may vary depending on the type of coffee (*espresso*, *lungo*, *cappuccino*, etc.), the size of the cup and even the type of coffee beans (and therefore, the amount of caffeine in a cup of coffee can range from ~50 to over 400 mg), one average cup of coffee contains a dose ~100 mg of caffeine [15]. Coffee is also a complex matrix of hundreds of compounds including but not limited to caffeine. Although the intake of a cup of coffee corresponds to several factors, including perceived health benefits, taste and social interrelationship [16], in the field of sports, consuming coffee mainly responds to the aim of enhancing athletic performance, recovery, and fat loss [17]. The use of coffee as an alternative to caffeine supplementation in a capsule has not been widely researched, especially in the field of exercise performance [18], likely because of the difficulty of controlling the dose of caffeine ingested and the potential inferences of other coffee components on the caffeine-derived benefits. Although less numerous than the researchers with caffeine, there is evidence of the benefits of coffee enhancing endurance cycling performance [11,19,20]. Additionally, there a few investigations aimed to determine if one can obtain similar ergogenic benefits from caffeine ingested in the form of coffee or from a capsule containing pure anhydrous caffeine when the dose of caffeine is matched [21,22]. These investigations are particularly interesting because they may help cyclists to decide on what form of caffeine administration is better for their performance-enhancing supplementation protocols before exercise. Interestingly, coffee was equally effective in enhancing sports performance than the caffeine administered in a capsule in two studies that used cycling activities [21,22,23]. However, in a study with a similar protocol performed on runners [24], only the ingestion of caffeine in the form of a capsule increased endurance running performance. These authors suggested that the ergogenic benefits of caffeine should not be extrapolated to coffee even when the dose of caffeine is matched as there may be some components of coffee that counteract the actions of caffeine. So, there is still a debate regarding whether coffee is an ergogenic aid for enhancing endurance performance.

On the other hand, most of the literature on the effects of caffeine on cycling performance has been tested in the laboratory. For example, in the systematic review by Southward et al. [25] about the effect of acute caffeine ingestion on endurance performance, 35 out of the 45 studies included assessed the effect of caffeine on cycling activities. However, all of them used a cycle ergometer in a controlled laboratory setting. While the data obtained in the laboratory can be transferred to cycling competitions to infer that endurance performance can be improved, it is difficult to interpret if this potential “physiological” benefit obtained with caffeine may be transferred to enhance performance in cycling situations with a complex technical component. Again, there is evidence of the benefits of caffeine to enhance performance during sports competitions with a certain technical component. For example, Potgieter et al. [26] found a reduction of 1.3% in the time to complete an Olympic-distance triathlon race. Additionally, MacIntosh et al. [27] found a 1500 m swimming time in a swimming race with 6 mg/kg body weight. However, there has been no research conducted to ascertain whether caffeine is ergogenic in a real cycling competitive context with the high demands of technical abilities.

Therefore, the aim of this study was to examine the effects of caffeine intake, via soluble coffee, on cross-country cycling performance in recreational cyclists. We selected this cycling discipline as cross-country cycling requires different demands, such as aerobic endurance, muscular endurance and technical ability [28]. According to Impellizzeri and Marcora [29], the majority of a race is undertaken at an intensity very close to that of the anaerobic threshold, but some sectors require the contribution of anaerobic pathways, particularly during uphill sections. Additionally, in cross-country cycling, there are areas of lower aerobic intensity, corresponding to downhill sections in which the “physiological attributes” of the cyclist are less relevant and the technical ability becomes a key performance factor. The main hypothesis was that acute intake of caffeine via coffee would improve performance in the cross-country cycling time trial. 

## 2. Materials and Methods

### 2.1. Participants

The sample consisted of 11 (men = 9; women = 2) young, healthy and physically active participants (Table 1). All of them were recreational cyclists with cross-country/mountain biking experience of at least 3 years. The sample size (estimated at a minimum of 11 individuals) was calculated based on a medium effect size of caffeine (0.78 Cohen’s units), based on a previous study that reported improvements in aerobic performance with 3 mg/kg body weight of caffeine in a laboratory-developed cycling time trial [7]. G*Power software (v.3.1.9., Düsseldorf, Germany) was used for sample size calculation, considering that a crossover investigation with two experimental trials was designed, aiming for a statistical power of 75% and an alpha error of 0.05, for a paired-sample *t*-test. We recruited both men and women until reaching the sample size required as men and women obtained comparable ergogenic benefits from caffeine intake during aerobic [30] and anaerobic cycling activities [31].

Participants met the following inclusion criteria: (a) age between 18 and 30 years; (b) stable maintenance of their training routines during the duration of the study; and (c) in women, the regular duration of their menstrual cycle for the previous 2 months. Participants were excluded if they: (a) had any cardiovascular or metabolic disease; (b) had suffered any type of musculoskeletal injury in the previous two months; (c) were mild-to-high consumers of caffeine represented by a mean daily intake of >2.99 mg/kg/day; (d) were smokers; (e) used supplementation or medication during the study; or (f) for women, used oral contraceptives or had menstrual disorders associated with menstruation. Prior to the start of the study, all participants were informed about the experimental procedures and risks and signed an informed consent form. The study was approved by an Institutional Review Board. Habitual caffeine intake was measured by using a modified version of the validated questionnaire by Bühler et al. [32], and the status was obtained using the categories suggested by Filip et al. [33].

### 2.2. Experimental Design

A randomised, single-blinded and counterbalanced crossover experimental design was conducted. Each subject participated in 4 trials: 2 familiarisation trials with no beverage intake and 2 identical experimental trials after the ingestion of a beverage that included a 13.90 km cross-country cycling time trial simulating a competition. All trials were separated by a period ranging from 48 h and one week to allow caffeine wash-out and recovery. In the first familiarisation trial, participants completed the course with one investigator at a relaxed pace to ensure learning of the route, particularly during the most technically difficult sections. We performed this initial trial as this is a customary practice of cross-country cyclists before the competition. In the second familiarisation trial, participants completed the course at maximum intensity without the intake of any beverage. In the third and fourth trials, participants randomly ingested (a) a cup of soluble caffeinated coffee (Nescafé Classic, Nestlé, Vevey, Switzerland) adjusted to provide 3 mg/kg body mass of caffeine (CAF), or (b) a cup of decaffeinated soluble coffee (Nescafé Decaffeinated, Nestlé, Switzerland) to simulate a placebo (PLA; 0.04 mg/kg of caffeine). For each participant, the amount of caffeinated/decaffeinated coffee was dissolved in 250 mL hot water and provided in an opaque drinking bottle approximately 60 min before the onset of the cross-country cycling time trial. These doses of coffee for each participant were calculated considering that 100 g of a prepared soluble coffee (Nescafé Classic, Nestlé, Switzerland) contains 3900 mg of caffeine [34]. Participants had 10 min to ingest the coffee and an investigator verified the entire ingestion of the beverage to assure intake of the treatment. The dose of 3 mg/kg of caffeine was chosen because it has been shown to be sufficient to elicit the ergogenic effects of caffeine on cycling endurance activities in laboratory settings [25]. The experimental trials were conducted at the same time of day to avoid the effects of circadian rhythms on the results of the study. Both experimental trials were carried out under similar weather and route conditions for each participant. The trials were completed between April and May 2023 with ambient conditions between 18 and 25 °C and humidity between 35 and 65%.

### 2.3. Experimental Protocol

Twenty-four hours before each experimental trial, participants were required to refrain from strenuous exercise and adopt a similar diet mimicking a pre-competition day. Before the first trial, participants completed a questionnaire about their food intake 24 h before the test and this questionnaire was used to replicate the meals in the second trial. They were also required to avoid alcohol, caffeine, tobacco and other stimulants 24 h before each trial and abstention from these substances was verified through diaries. On testing days, participants arrived at the cross-country course, located in Casa de Campo (Madrid) with their bikes and wearing cycling shorts and their MTB shoes. One investigator checked that the parameters of the bike were appropriate, including tyre pressure and cycling seat height. Afterwards, participants drank the assigned beverage and rested for 45 min to ensure substance absorption [8]. During this time, a Global Positioning System (GPS) device (Vivoactive 3, Garmin, Olathe, KS, USA) was adjusted to the handlebar of the bike and a heart rate monitor was attached to the participant’s chest (H10, Polar, Kempele, Finland). Then, participants performed a 5 km warm-up which lasted approximately 15 min to allow the cycling time trial to start just 60 min after coffee ingestion. Once the warm-up was completed, the GPS parameters were calibrated and the GPS signal was activated for one minute for accurate data recording. At this point, the importance of performing the test with maximum effort was highlighted. Then the participants started the time trial and the time data recording started automatically. During the tests, performance parameters such as peak speed, finishing and lap times and average heart rate were measured. Immediately after finishing the test, participants were asked to indicate their rating of perceived exertion (RPE) according to the Borg scale ranging from 6 to 20 points, where 6 points meant no effort at all and 20 meant maximum effort [35]. At the same time, they were also asked verbally what they thought they had ingested, with a choice of 3 options: “Caffeine”, “Placebo” or “I don’t know” to evaluate the quality of the blinding process using the Bang index [36].

#### Course Characteristics

**Figure 1:**
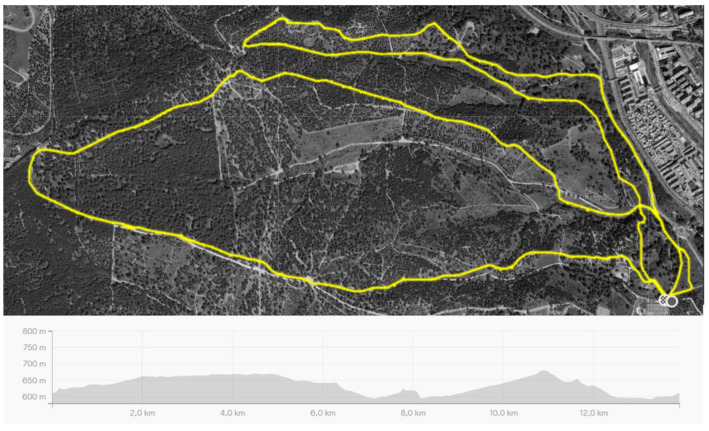
Circuit route and altimetric profile. The yellow line indicates the path of the course.

The cross-country cycling course was designed at Casa de Campo Park in Madrid, an area where races belonging to the professional mountain bike racing circuit had previously been held. The circuit in this experiment had a total distance of 13.90 km and 208 m of cumulative positive ascent. The circuit was designed to include both technically and physically demanding sections to make it as similar as possible to a real cross-country cycling race (Figure 1).

**Figure 2:**
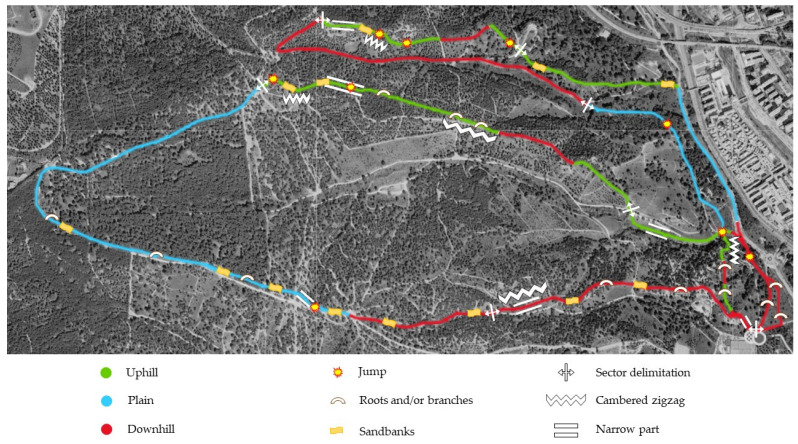
Graphical representation of the technical characteristics of the course by sector.

The circuit was figure-of-eight-shaped, and it was divided into two laps with different itineraries. The circuit had eight different sectors, which allowed for a more exhaustive analysis of the cycling performance. Technical areas with significant slopes (both positive and negative), narrow paths with constant changes of direction, areas with sandbanks, branches, roots and jumps and flat ground were present in the course. Figure 2 depicts a more in-depth description of the course and its eight sectors. The technical difficulty of each area was determined by the three experienced and independent cross-country cyclists who completed the course twice and were allowed to catalogue the difficulty of each sector using a 1-to-5-point scale. Data on the assessment of the technical difficulty of each sector is presented in Table 2. The value of the technical difficulty of each sector was reached by consensus of these three experienced cyclists. Data on the gradient ascent/descent section were obtained from Strava https://www.strava.com/ (United States).

### 2.4. Analysis

Statistical analysis was performed using SPSS software version 27.0 for Windows (SPSS Inc., Chicago, IL, USA). In the analysis, men and women were included in the same group as similar ergogenic responses to caffeine were previously found between men and women [30]. First, the Shapiro–Wilk test was performed to test for normality for each variable (*p* > 0.05). Subsequently, a paired-sample Student’s *t*-test was performed to analyse differences in normally distributed variables between caffeinated coffee (CAF) and placebo/decaffeinated coffee (PLA). The significance level was set at *p* < 0.050. For not normally distributed variables, a non-parametric paired-sample Wilcoxon test was used. The magnitude of the effect size was calculated with Cohen’s *d* and interpreted according to the following scale [37]: small (0.20–0.49), medium (0.50–0.79) and large (≥0.80). Bang’s index was calculated to assess whether subjects were able to identify in which trial they had ingested the caffeinated and decaffeinated coffee [36].

## 3. Results

### 3.1. Complete Course

Participants were unable to identify which type of coffee they had consumed in each of the trials. Of the 11 participants, three could not differentiate what they had consumed in either of their attempts, four of them correctly guessed the type of coffee assigned in each trial and the remaining four incorrectly guessed the types of coffee. As a result, the Bang index for the caffeinated coffee situation was 0 and 0 for the placebo/decaffeinated coffee (Table 3). These values indicate perfect blinding for the experiment.

**Figure 3:**
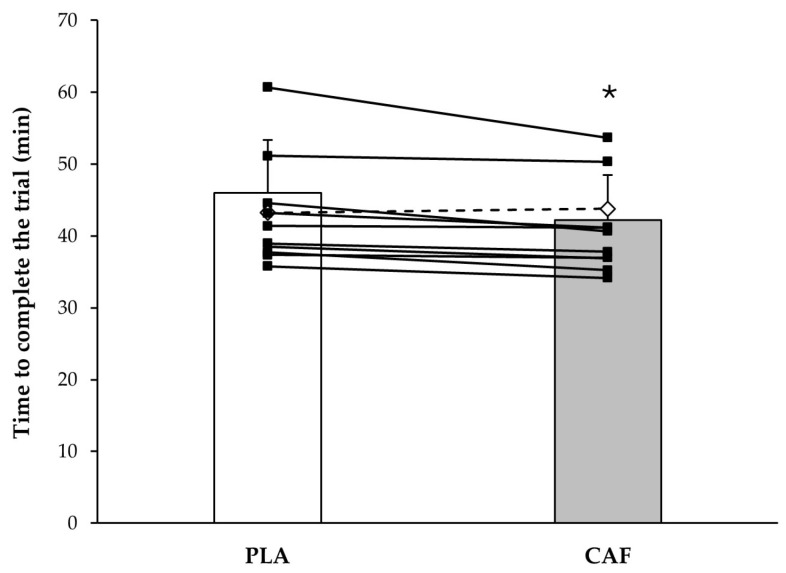
Time to complete a cross-country cycling time trial after consuming 3 mg/kg body mass of caffeine in the form of coffee or a placebo in the form of decaffeinated coffee. (*) Significant differences between caffeine and placebo conditions (*p* < 0.050). CAF = caffeinated coffee; PLA = placebo/decaffeinated coffee. The columns represent the group’s average with each treatment and the whiskers represent a standard deviation of 1. The solid lines represent individual responses for participants with lower time trials with CAF than with PLA. The dashed lines represent individual responses for participants with higher time trials with CAF than with PLA.

The time to complete the cross-country time trial was significantly shorter with the caffeinated coffee (CAF) than with the decaffeinated/placebo (PLA) coffee (43.20 ± 7.35 vs. 41.17 ± 6.18 min; *t* = 3.137; *d* = 0.300; *p* = 0.011). There was a significant reduction in the time to complete the first lap with CAF vs. PLA (20.52 ± 2.70 vs. 21.74 ± 2.76 min; *t* = 3.654; *d* = 0.446; *p* = 0.004) with no statistically significant differences between conditions for the second lap (20.65 ± 3.60 vs. 21.40 ± 4.80 min; *z* = 1.334; *d* = 0.178; *p* = 0.182; Figure 3).

Mean heart rate during the trial was higher in CAF than in PLA (169 ± 6 vs. 162 ± 13 bpm; *t* = 2.363; *p* = 0.046; *d* = 0.788). However, there were no significant differences between conditions in the RPE values obtained at the end of the exercise (16 ± 1 vs. 16 ± 2 a.u.; *d* = 0.061; *p* = 0.676).

### 3.2. Course Sectors

In comparison to PLA, CAF significantly reduced the time to cover sectors “1”, “2”, “3” and “4” (Table 4). No statistically significant differences between conditions were found in the times of the remaining sectors (*p* ≥ 0.05). No statistically significant differences were found in heart rate between conditions for any of the eight sectors that composed the course (Table 5; *p* > 0.05). Significantly higher maximum speeds were observed in sectors “1”, “2” and “3” for the CAF compared to the PLA condition, exceeding on average 2.05 ± 2.63 km/h, 1.80 ± 1.83 km/h and 1.96 ± 2.10 km/h, respectively for CAF over the PLA trial. 

## 4. Discussion

The present study sought to explore the effect of caffeine delivered via soluble coffee on cross-country cycling performance in recreational cyclists. The previous literature on this topic habitually used caffeine anhydrous in capsules as a form of caffeine administration and tested the effect of this substance in cycling tests performed on a cycle ergometer in a laboratory [25]. This investigation is novel because it determines the potential physiological benefits of caffeine in a more ecologically valid scenario for cross-country cyclists represented by a simulated cross-country cycling competition/time trial on a course with sectors of different technical difficulty while caffeine is administered as coffee. The main finding of this investigation was that 3 mg/kg of caffeine intake in the form of coffee reduced the time to finish the cycling trial by 4.93 ± 4.39% (~2 min less for a 43 min cycling time trial). The overall lower time to complete the trial was produced by lower times in sectors of varying technical difficulty. Additionally, the caffeine in the form of coffee increased the mean heart rate during the trial without affecting RPE at the end of the exercise. This indicates that caffeine enhanced endurance, expressed by a lower time to complete the distance and the capacity to sustain a higher mean heart rate during the trial while maintaining participants’ perceived fatigue. The benefits of caffeine were observed mainly during the first half of the course, irrespective of the technical difficulty of the sector observed in finishing times and maximum speed at several course sectors, particularly in the first four.

According to the scientific literature, caffeine is ergogenic when ingested in doses between 3 and 6 mg/kg with minimal differences in the magnitude of the benefit within this dose range [4,38]. This caffeine dose range also applies to cycling activities as evidence suggests a benefit of caffeine to enhance endurance cycling performance when ingested in a dose between 3 and 6 mg/kg [6,25]. The dose of caffeine used in the current study was 3 mg/kg body mass, which is in the lower range of dosing with established ergogenic benefits. Although the current experiment included only one dose of caffeine, there is evidence suggesting that it may be difficult for doses < 3 mg/kg of caffeine to yield such benefits during endurance cycling activities [39]. In the present study, soluble coffee was used as an alternative to direct caffeine supplementation. The observed effects could be attributed to caffeine since, as concluded by Hodgson et al. [21], the use of soluble coffee with caffeine may be just as ergogenic as caffeine when the dose of caffeine is appropriate. Although coffee has hundreds of compounds [17], it seems that the other substances inherent to coffee such as chlorogenic acids do not interfere with the ergogenic properties of caffeine. In a systematic review by Higgins et al. [23], the authors concluded that coffee may be ergogenic when the amount of coffee ingested provides between 3 and 8 mg/kg of caffeine. Collectively, all this information suggests that caffeine is an ergogenic substance to enhance endurance cycling performance when ingested in a dose of at least 3 mg/kg. The method of caffeine administration seems less relevant once the minimum dose of caffeine is ingested. Therefore, caffeinated coffee may be used as a safe alternative to anhydrous caffeine to improve endurance cycling performance. In the opinion of the authors, the use of soluble coffee may be recommendable over other types of coffee because it allows an accurate calculation of the dose administered.

Several previous research studies investigated the ergogenic effect of caffeine in a cycling time trial. Most studies were conducted on a cycle ergometer, in a controlled and stable laboratory environment [7,11]. The most commonly used distances ranged from 10 to 20 km, although time to complete a given amount of work is another experimental approach to test the effect of caffeine on endurance cycling performance [25]. Additionally, Astorino et al. [40] demonstrated that the effect of caffeine to enhance cycling performance is repeatable, as they found that 5 mg/kg of caffeine produced similar benefits (over a placebo) when given on two different days. Last, it has also been demonstrated that the ergogenic effects of caffeine on cycling endurance are independent of participants’ fitness level, as Astorino et al. [41] found consistent performance improvements in endurance athletes and active individuals. All this evidence has settled the current notion of considering caffeine as an ergogenic substance with the capacity to enhance cycling performance by 2.3–3.2% [5,25]. However, the findings of these studies are sometimes difficult to transfer to real cycling scenarios as most cycling competitions require, in addition to excellent endurance capacities, the development of technical abilities. So, it may be assumed that in some complex competitions such as cross-country cycling, the potential benefits of caffeine on endurance performance may not be obtained as, in some sectors of a race, a better physiological status cannot be translated into a better performance. The current investigation was designed for a course of cross-country cycling with sectors of different technical difficulties to test this hypothesis. Interestingly, the effect of caffeine on cycling performance (a 4.3% reduction in the time to complete the trial) was similar to previous studies performed in the laboratory, while the effect of caffeine was present in the four first sectors of the course despite having different technical demands. For example, caffeine reduced the time to complete the sector with the greatest technical difficulty (Sector “3”, with four out of five points of maximal technical difficulty). In this case, it seemed that caffeine was ergogenic to enhance performance during the first half of the race while its effect was diminished during the second half. This is the first investigation that demonstrates the benefits of caffeine during a complex cycling competition developed in a real scenario. Taken together, all this information suggests that caffeine ergogenicity may be present during real cycling competitions that require cyclists’ technical ability.

Still, caffeine was not ergogenic in some sectors of the cross-country cycling course. This is likely because cross-country is a particular cycling activity with complex sections where a higher cycling power is not always translated into better cycling performance. For example, it has been found that pedalling on a downhill section of a cross-country competition was not always necessary to improve performance [42]. Similarly, Impellizzeri and Marcora [29] reported that the power generated during a cross-country cycling event did not necessarily correlate with better performance. This difference in cycling power vs. cycling performance may be explained by the fact that those who took longer to complete a cross-country cycling competition are sometimes able to generate more power than those who performed better, because of the important role played by technique and its relationship with economy. During a cross-country competition, the power developed is largely influenced by the characteristics of the terrain [29]. These characteristics constantly impact performance-related aspects, requiring, for example, less gearing on the bike and more cadence to make progress on climbs with very little grip and the wheel spinning without making progress [43]. In short, the technical characteristics of each sector likely had a strong influence on physiological responses during the time trial. In this regard, it is possible that the better physiological performance with caffeine could not be transferred in some sectors where the time to complete it was more associated with technical ability than power generation. However, the findings of the present study were inconclusive about the effectiveness of caffeine in improving the technical ability of mountain bikers and further research is required to address this topic.

The current investigation has several limitations that should be discussed to understand the potential application of the outcomes. The first limitation is that we used only one dose of caffeine (i.e., 3 mg/kg) in the form of coffee, and we tested this substance in a cross-country competition. Still, more investigation is needed to understand the potential benefits of lower and higher doses of caffeine in other cycling disciplines, such as road cycling, track cycling or BMX. Although lower doses of caffeine have been tested as ineffective in enhancing cycling performance [39], and doubling the dose from 3 to 6 mg/kg has been ineffective in enhancing the magnitude of the caffeine-derived benefits [6] in the laboratory, further investigations should test the dose response to caffeine in real cycling scenarios. Second, we used a sample of recreational cyclists, and the effect of caffeine on high-performance cyclists should be further explored. Third, we did not measure circulating caffeine concentration to confirm caffeine abstention prior to the trials and to quantify caffeine availability following ingestion during the trials. However, we confirmed caffeine abstention through dietary diaries and used a 60 min period for caffeine absorption between intake and the start of the time trial to assure near-to-peak serum caffeine concentration during the trials [44]. Fourth, we used a sample of cyclists with low daily caffeine intake. As chronic ingestion of caffeine may reduce the potential effect of acute caffeine intake on cycling performance both at the ventilatory threshold [45] and at VO_2_max [46], the data of this study should only applied to individuals with a low habitual caffeine intake. Fifth, the number of women in our study was not enough to perform a sub-analysis by sex with the appropriate statistical power. Last, we provided data per sector to understand the potential effect of caffeine depending on the technical difficulty of the cross-country cycling section. However, all the sectors contained a mix of sections with uphill and downhill sections of various technical difficulties. In order to evaluate specifically the effect of caffeine intake on the technical abilities of cross-country cyclists, future investigations should measure the effect of caffeine on isolated sections with differential technical situations (e.g., rock garden vs. mud vs. gravel).

## 5. Conclusions

In conclusion, 3 mg/kg of caffeine consumed in the form of soluble coffee 60 min before the start of a 13.90 km cross-country time trial reduced the time needed to complete the distance in recreational cyclists. The better physical performance with the caffeinated coffee was accompanied by a higher mean heart rate (as a sign of higher exercise intensity) but without affecting the rating of perceived exertion after the trial. The ergogenic effect of caffeine was present in sectors with different technical complexity, mainly in the first half of the trial. These outcomes suggest that acute caffeine intake can be effectively employed during a cross-country cycling race to enhance overall cycling performance. Results of the present study can be applied by recreational mountain bike/cross-country cyclists with a low habitual caffeine intake, who could obtain a ~4% performance benefit through the intake of caffeinated coffee before the race. As a practical application, cyclists planning to use coffee as a source of caffeine before exercise should use a type of coffee that allows an accurate calculation of the absolute dose of caffeine ingested. This is important as the amount of caffeine in a cup of coffee may greatly vary depending on the type of coffee, the size of the serving and the type of beans. The use of soluble coffee before exercise may be more recommendable than espresso coffee as the amount of caffeine can be calculated from the grams of soluble coffee dissolved in water.

## Figures and Tables

**Table 1:** Main characteristics of the participants expressed in means, standard deviations (SD) and ranges.

	Mean (SD)		
	Total	Men	Women
Age	22 (3)	20 (3)	22 (4)
Min.	18	18	19
Max.	27	27	24
Height (cm)	177.2 (8.2)	180.1 (5.6)	164.0 (0)
Min.	164.0	173.5	164.0
Max.	189.0	189.0	164.0
Body mass (kg)	70.91 (9.15)	73.20 (8.18)	60.55 (6.43)
Min.	56.00	62.80	56.00
Max.	84.00	84.00	65.10
Body mass index (kg/m^2^)	22.59 (2.54)	22.61 (2.73)	22.51 (2.39)
Min.	19.66	19.66	20.82
Max.	26.79	26.79	24.2
Resting heart rate (bpm)	60 (11)	57 (9)	72 (16)
Min.	43	43	60
Max.	83	73	83
Daily caffeine intake (mg/kg/day)	0.79 (0.64)	0.72 (0.70)	1.11 (0.01)
Min.	0.12	0.12	1.10
Max.	2.19	2.19	1.12

**Table 2:** Detailed description of the characteristics of each course sector.

Sector	Description	Technical Difficulty				
1	Intense uphill (32.1% gradient peak) with roots and sandbanks on the ground.	1	2	3	4	5
2	Moderate uphill (~4% gradient), linking with a path (gradient between −2.5 and 2.5) that starts with a jump. There are several roots and sandbanks, but the route is relatively straight and easy.	1	2	3	4	5
3	Combination of fast curves and steep jumps in the most complex section of the circuit. The negative gradient is approximately −5 to −9%. The path turns into a short uphill section followed by a descent with a −16.6% negative gradient peak.	1	2	3	4	5
4	This section starts with a very narrow descent (−6% gradient) which turns into a steady uphill section with minimal technical difficulty (gradient between 2.5 and 7.2%).	1	2	3	4	5
5	Downhill section that connects with a relatively complex descent with short curves with a gradient of −17.0–35.8%.	1	2	3	4	5
6	Climb on a wide track without technical difficulty. Constant gradient of ~4.5% with a peak gradient of 12%.	1	2	3	4	5
7	Steep descent (between −12 and −17.6%) with 2 complex turns followed by an uphill section (9.4% gradient) then descends again with a −20.9% negative slope.	1	2	3	4	5
8	Continuation of the descent of the previous section followed by an easy but narrow uphill section of between 2.5 and 10% to the finish line.	1	2	3	4	5

The dashed box indicates the technical difficulty of each course sector.

**Table 3:** Bang index for the estimation of the quality of the blind.

Trials	Answer		
	Caffeine	Placebo	Do Not Know
Caffeine	4	4	3
Placebo	4	4	3
Total	8	8	6
Bang index	0.0	0.0	

The figures for “caffeine” and “placebo” trials (in rows) indicate the number of participants that responded what they believed they had ingested in each trial, between the three possible answers (in columns). With this data, the Bang index was calculated. Bang index values may vary between −1 and 1 for each experimental situation. The closer to 0, the higher the quality of the blinding.

**Table 4:** Time by sector (in min) during a cross-country cycling time trial after consuming 3 mg/kg body mass of caffeine in the form of coffee or a placebo in the form of decaffeinated coffee. (*) Significant differences between caffeine and placebo conditions (*p* < 0.050). CAF = caffeinated coffee; PLA = placebo/decaffeinated coffee.

	CAF	PLA	*t*	*d*	*p*
Sector 1	4.22 ± 0.77	4.59 ± 0.70	4.571	0.498	0.001 *
Sector 2	9.34 ± 1.20	9.81 ± 1.22	3.152	0.386	0.010 *
Sector 3	4.04 ± 0.64	4.52 ± 0.73	3.601	0.701	0.005 *
Sector 4	2.64 ± 0.36	2.82 ± 0.37	3.215	0.475	0.009 *
Sector 5	3.64 ± 0.91	3.62 ± 1.03	0.044 ^z^	0.023	0.965
Sector 6	8.91 ± 1.57	9.21 ± 1.73	1.956 ^z^	0.182	0.050
Sector 7	2.78 ± 0.51	3.03 ± 0.96	1.476	0.346	0.171
Sector 8	5.50 ± 0.80	5.69 ± 1.11	1.406	0.201	0.190

Mean values ± standard deviation; t-value (*t*); effect size (*d*); and significance level (*p*) are expressed in the table. ^z^ indicates Wilcoxon’s test value for the non-normally distributed data. * Statistically significant differences between CAF and PLA conditions (*p* < 0.05).

**Table 5:** Heart rate (in bpm) and peak speed (in km/h) per sector during a cross-country cycling time trial after consuming 3 mg/kg body mass of caffeine in the form of coffee or a placebo in the form of decaffeinated coffee. (*) Significant differences between caffeine and placebo conditions (*p* < 0.050). CAF = caffeinated coffee; PLA = placebo/decaffeinated coffee.

	CAF	PLA	*t*	*d*	*p*
Heart rate (bpm)					
Sector 1	129 ± 18	129 ± 22	0.133 ^z^	0.005	0.894
Sector 2	170 ± 11	159 ± 17	1.512 ^z^	0.657	0.130
Sector 3	171 ± 11	168 ± 11	0.714 ^z^	0.310	0.475
Sector 4	171 ± 12	166 ± 12	1.601 ^z^	0.535	0.109
Sector 5	173 ± 8	169 ± 10	1.602 ^z^	0.436	0.109
Sector 6	173 ± 10	171 ± 10	0.313 ^z^	0.232	0.754
Sector 7	172 ± 10	171 ± 7	1.072 ^z^	0.195	0.284
Sector 8	173 ± 8	171 ± 9	1.386 ^z^	0.511	0.166
Peak speed (km/h)					
Sector 1	37.25 ± 6.16	35.2 ± 4.6	2.575	0.380	0.028 *
Sector 2	32.85 ± 3.60	31.08 ± 3.53	3.202	0.497	0.009 *
Sector 3	47.38 ± 8.24	45.42 ± 9.34	3.084	0.223	0.012 *
Sector 4	36.44 ± 3.64	36.35 ± 3.96	0.157	0.024	0.879
Sector 5	38.69 ± 5.46	37.87 ± 6.76	1.156 ^z^	0.134	0.258
Sector 6	24.77 ± 3.49	25.29 ± 3.83	0.704	0.141	0.497
Sector 7	44.05 ± 6.74	43.87 ± 7.52	0.154	0.024	0.881
Sector 8	41.70 ± 8.94	42.30 ± 9.30	0.749	0.066	0.471

Mean values ± standard deviation; *t*-value (*t*); effect size (*d*); and significance level (*p*) are expressed in the table. ^z^ indicates Wilcoxon’s test value for the non-normally distributed data. * Statistically significant differences between CAF and PLA conditions (*p* < 0.05).

## Data Availability

The data presented in this study are available on request from the corresponding author. The raw data supporting the conclusions of this article will be made available by the authors on request.
